# Hammerhead Ribozymes: True Metal or Nucleobase Catalysis? Where Is the Catalytic Power from?

**DOI:** 10.3390/molecules15085389

**Published:** 2010-08-06

**Authors:** Fabrice Leclerc

**Affiliations:** Laboratoire ARN-RNP Maturation-Structure-Fonction, Enzymologie Enzymologie Moléculaire et Structurale (AREMS), UMR 7214 CNRS-UHP, Nancy Universités, Faculté des Sciences et Technologies, Bld. des Aiguillettes, BP 70239, 54506, Vandoeuvre-lès-Nancy, France; E-Mail: Fabrice.Leclerc@uhp-nancy.fr; Tel.: +33-(0)-383684317; Fax: +33-(0)-383684307

**Keywords:** ribozyme, RNA catalysis, metal ion, hammerhead, nucleobase, self-cleaving, self-splicing, evolution

## Abstract

The hammerhead ribozyme was first considered as a metalloenzyme despite persistent inconsistencies between structural and functional data. In the last decade, metal ions were confirmed as catalysts in self-splicing ribozymes but displaced by nucleobases in self-cleaving ribozymes. However, a model of catalysis just relying on nucleobases as catalysts does not fully fit some recent data. Gathering and comparing data on metal ions in self-cleaving and self-splicing ribozymes, the roles of divalent metal ions and nucleobases are revisited. Hypothetical models based on cooperation between metal ions and nucleobases are proposed for the catalysis and evolution of this prototype in RNA catalysis.

## 1. Introduction

**Figure 1:**
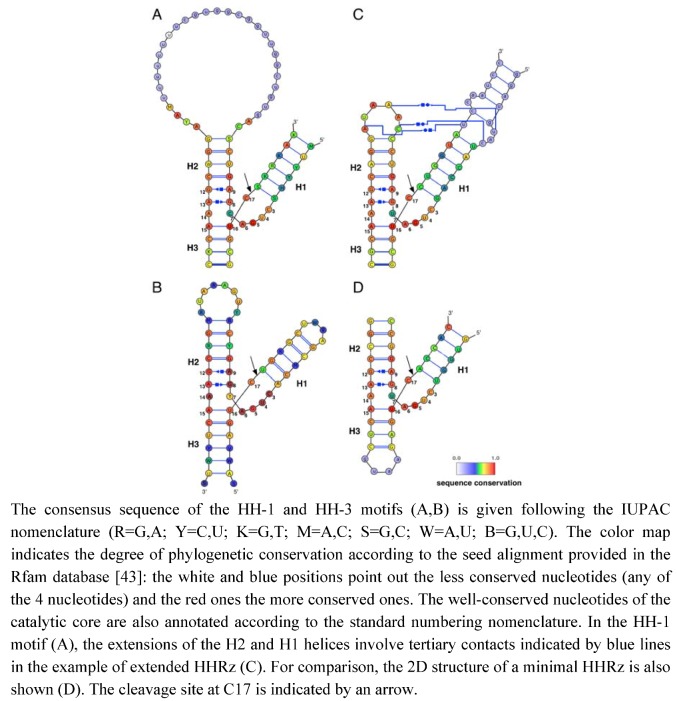
HHRz motifs and variants. (A) HH-1 motif (Rfam ID: RF00163). (B) HH-3 motif (Rfam ID: RF00008). (C) HH-1 extended motif from *Schistosoma mansoni* [42]. (D) HH-1 minimal motif truncated from the lucerne transient streak virus (PDB ID: 301D [23]).

Hammerhead ribozymes are commonly presented as the smallest natural ribozymes and have been considered as a prototype of RNA catalysts and RNA metalloenzymes [1]. Previously, much larger RNA molecules such as RNase P [2] and group I introns [3] were shown to carry a catalytic activity. On the other hand, the hammerhead ribozyme (HHRz) is the smallest member among the nucleolytic ribozymes [4,5,6]. The HHRz is a three-way junction RNA motif including three base-paired helices joined (by single-stranded linkers) at a highly conserved central loop core matching the catalytic core of the ribozyme. The RNA fold is preserved even if the three distinct helices H1, H2 and H3 are connected in a different order in the RNA sequence. Thus, we refer to the HH-1, HH-2 or HH-3 motifs whether the terminal helix with open 5’ and 3’ ends is H1, H2 or H3 helix respectively [7] (Figure 1). This motif diversity is nicely illustrated in naturally occurring HH-1 and HH-3 motifs associated with functional HHRz. The HH-1 and HH-3 motifs are found for example in the Smalpha repetitive DNA of *Schistosoma mansoni* and in the Tobacco ringspot virus [7,8], respectively. Originally discovered in such plant viruses, the HHRz appears to be widespread in the genomes of phylogenetically distant organisms [9,10,11,12,13]. These evolutionary data and those collected from artificial evolution suggest that the HHRz is an example of convergent evolution providing a simple solution to RNA catalysis by self-cleaving in a small RNA motif [7,14].

The site-specific phosphodiester bond cleavage is a phosphoryl-transfer reaction performed through the isomerization of a 5’,3’-diester. The reaction generates 5’-hydroxyl and 2’-3’-cyclic phosphate termini: a chemical signature from self-cleaving ribozymes, a family of ribozymes also including the hairpin, HDV, glmS or *vs.* ribozymes [15]. In contrast, the phosphodiester cleavage reaction taking place in self-splicing ribozymes generates 5’-phosphate and 3’-hydroxyl termini due to the participation of different attacking nucleophiles. In group I introns, the attacking nulceophile is an exogenous 3’-hydroxyl group (guanosine as cofactor) while it is an endogenous 2’-hydroxyl group (intronic adenosine) in group II introns. In both self-cleaving or self-splicing ribozymes, the reaction proceeds via an S_N_2(P) or ‘in-line’ mechanism in which the attacking nucleophile is aligned with the phosphorus atom and the leaving group oxygen of the phosphate group which adopts a trigonal bipyramidal geometry [16,17,18]. In the case of self-cleaving ribozymes, the trigonal bipyramidal pentacoordinated phosphate group is formed during the transition state by the juxtaposition around the phosphorus of the two aligned oxygens from the endogenous attacking nucleophile (2’-OH) and leaving group (5’-OR) and the two non-bridging oxygens. The reaction proceeds via the same S_N_2(P)-type mechanism in both self-cleaving and self-splicing ribozymes as in uncatalyzed reactions in solution [19]. Nevertheless, the catalytic strategy in each family of ribozymes may differ about the requirement of divalent metal ions for catalytic and biological activity. The current data suggest that the self-splicing ribozymes operate as metalloenzymes whereas the self-cleaving ribozymes follow a general acid-base catalysis through the participation of "activated" nucleobases [15]. Thus, the overall mechanism in self-cleaving ribozymes is similar to that found in bovine pancreatic RNAse where the reaction proceeds via a general acid/base catalysis involving amino-acid side-chains (histidine residues) as catalysts [20]. A variety of protein and RNA enzymes also cleave phosphodiester bonds through a mixed general base/Lewis acid catalysis where metal ions can act as general base and/or Lewis acid [21]. Obviously, the catalytic strategy is the result of the molecular evolution of the ribozyme structure in particular in the catalytic core (Figure 1). Therefore, it is critical to have supporting structural data to understand how the catalytic site and its environment determines the catalytic strategy. 

The HHRz was the first ribozyme crystallized in a biologically-active form which provided a series of X-ray structures as possible snapshots along the reaction paths [22,23,24,25]. However, the experimental determination of RNA 3D structures remains particularly difficult. For this reason, most of the early efforts were focused on a "minimal" hammerhead ribozyme truncated in its H1 domain (Figure 1D). Several metal binding sites were found in the ground state conformation and in the conformational variants identified by crystallographic freeze-trapping before the cleavage reaction occurs [24,25]. Two metal ions are particularly close to the cleavage site in the 3D structures of the "minimal" HHRz [23]. Early models proposed for the detailed reaction mechanism from these structural data were essentially of two kinds: single-metal-ion models [26,27,28,29] or two-metal-ion models [21,30]. The qualitative single-metal-ion and two-metal-ion models were mostly based on a general acid/base catalysis and on a Lewis acid catalysis, respectively. However, quantitative models based on theoretical *ab initio* and DFT calculations actually supported a mixed general base/Lewis acid catalysis. In these models, the metal(s) plays both the roles of a general base (metal hydroxide) to activate the attacking nucleophile (2’OH) and that of a Lewis acid to stabilize the leaving group (5’OR) [29,31,32]. Finally, the metal-ion models essentially differed whether a single metal ion [29] or two metal ions can [31,32] act efficiently both as general base and Lewis acid at the same time or in a cooperative way. Anyway, they implicitly assumed the absence of major conformational changes in the structure of the ribozyme associated with the cleavage reaction. The structural data on the minimal HHRz supported a ‘local conformational rearrangement’ model at the catalytic site (C17 and neighboring nucleotides) for the transition from the ‘ground state’ to the ‘active’ conformation (consistent with the in-line mechanism). On the other hand, biochemical data obtained in solution supported a ‘global conformational rearrangement’ model. In particular, it would bring close to each other, through metal binding, two distant residues (9 and 17) which are almost 20Å apart in the ground state conformation. As result, new tertiary contacts would involve other remote residues. The comparison between the ‘minimal’ and ‘extended’ HHRz revealed the presence of sequence-specific interactions (between the loops capping helices H1 and H3) (Figure 1 C) associated with reaction rate enhancements [33,34,35]. In addition, the ‘extended’ HHRz has a weaker requirement of divalent metal ions for catalytic activity [34,36,37]. Besides, various and recent experimental data now support a model for general acid-base catalysis in the HHRz where divalent cations only play minor and structural roles. These data showed that: (1) the HHRz is partially active in presence only of monovalent cations replacing Mg^2+^ [24,38], (2) the pH-activity relationships of G8 and G12 variants are consistent with a nucleobase catalysis [39], (3) the chemical modifications of G8 and G12 are also consistent with nucleobase catalysis [40,41]. 

## 2. Results and Discussion

### 2.1. Metal Binding Sites in Self-Cleaving and Self-Splicing Ribozymes

**Figure 2:**
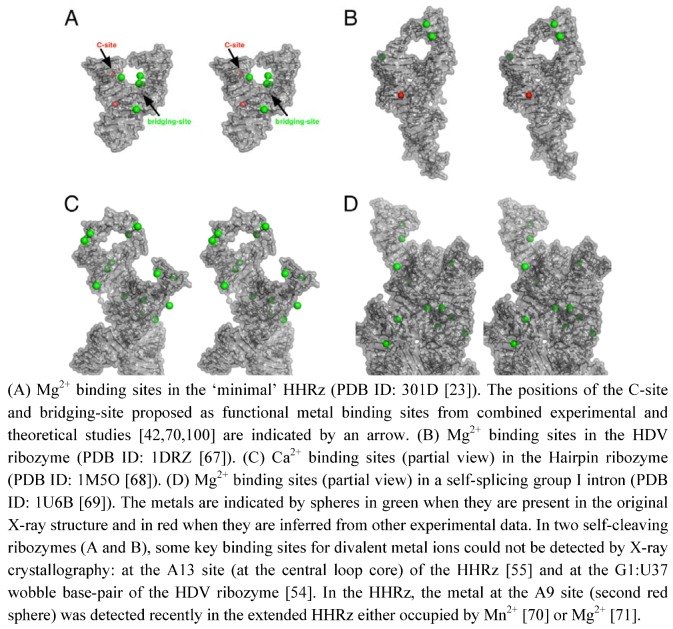
Stereo views of metal binding sites in the X-ray structures of self-cleaving and self-splicing ribozymes.

The role of metal ions in the structure and function of RNA has been known for a long time [44]. The requirement of metal ions for the folding and/or the stabilization of RNA tertiary structures show that metal ions play a major structural role in different ways. The metal binding can be: (1) nonspecific through “diffuse binding” [44] or “charge screening” [45] or (2) specific by outer-sphere or inner-sphere coordinations to RNA functional groups [44,45]. In proteins, metal binding sites are sculpted through well-defined coordinations to specific amino acid residues. Thus, metalloproteins are viewed in a static state as robust metal complexes with high- affinity metal-binding sites [46]. However, even in zinc finger proteins where the metal ion keeps the protein in the correct folding, metal exchanges occur in solution [47]. In RNA, some well-defined metal binding sites present in self-splicing ribozymes [48,49,50,51] are reminiscent to the protein sites. A striking metal binding similarity exist, for example, between group I introns [48] and nucleotidyl-transfer enzymes [52,53]. In contrast, some functional metal binding sites have been difficult to identify in self-cleaving ribozymes by X-ray crystallography (Figure 2). Some of them have been identified by other experimental approaches, for example in the HDV ribozyme [54] or in the minimal HHRz [55,56]. Sometimes, the representation of electrostatic profiles is also useful to predict the near location of bound metals [57]. Clearly, the metal ions seem to behave in a different way between self-splicing and self-cleaving ribozymes. In large-sized ribozymes, they are probably tightly bound metals and they play both major structural and catalytic roles. The self-splicing ribozymes that are compact and mostly globular can easily trap metal ions which remain deeply buried within the RNA scaffold (Figure 2 D). In small-sized ribozymes, the metal ions remain largely hydrated and less often coordinated to the RNA scaffold (Figure 2A-C). Those ribozymes require lower concentrations of divalent cations for folding. The metal ions seem much more mobile and they can be associated with minor and major conformational changes [58,59]. Whether the metal is involved in some induced conformational change or just redistributed afterwards remains to be elucidated.

The large number of donor atoms for metal-ion coordinations in nucleotides [45,60,61] may naturally confer a highly exchangeable behavior to metals in any ribozyme by the juxtaposition of multiple potential binding sites at the RNA surface. However, the ribozyme structures seem to have evolved in a different way using tightly bound metals in self-splicing ribozymes and more loosely bound metals in self-cleaving ribozymes. Thus, the structural and catalytic roles of metal ions are more clearly distinguishable in the folded self-splicing ribozymes. Assigning specific roles to metals in self-cleaving ribozymes is challenging because they may affect directly the chemical reaction itself or the RNA structure around the cleavage site and indirectly the reaction kinetics. According to the definition of a metal catalyst given by DeRose [62], such a metal ion "contributes to stabilizing the transition state" and is "associated with a group that changes bond order during the reaction". Nonetheless, this definition of metal ions as catalysts may be a bit restrictive considering that metal ions may control or modulate the catalytic activity in different ways. Even if they do not connect to the group that changes bond order during the reaction [61,63,64], the divalent cations can be involved in long-range electrostatic interactions and may have a significant impact on the catalytic activity while being far from the catalytic site [63]. So, one may prefer to distinguish between ‘specific’ catalytic metals, when they have short-range interactions (via inner- or outer-sphere coordination), from ‘nonspecific’ catalytic metals when they have longer range interactions with the residues of the catalytic site. Similarly, we could distinguish specific nucleobases that participate in bond making or bond breaking [65] from ‘nonspecific’ nucleobases (in a standard or ionized form [66]) that only take part in the catalytic site organization or in the binding of nucleotide or metal cofactors. 

### 2.2. Metal / Nucleobase in the HHRz Catalysis

**Figure 3:**
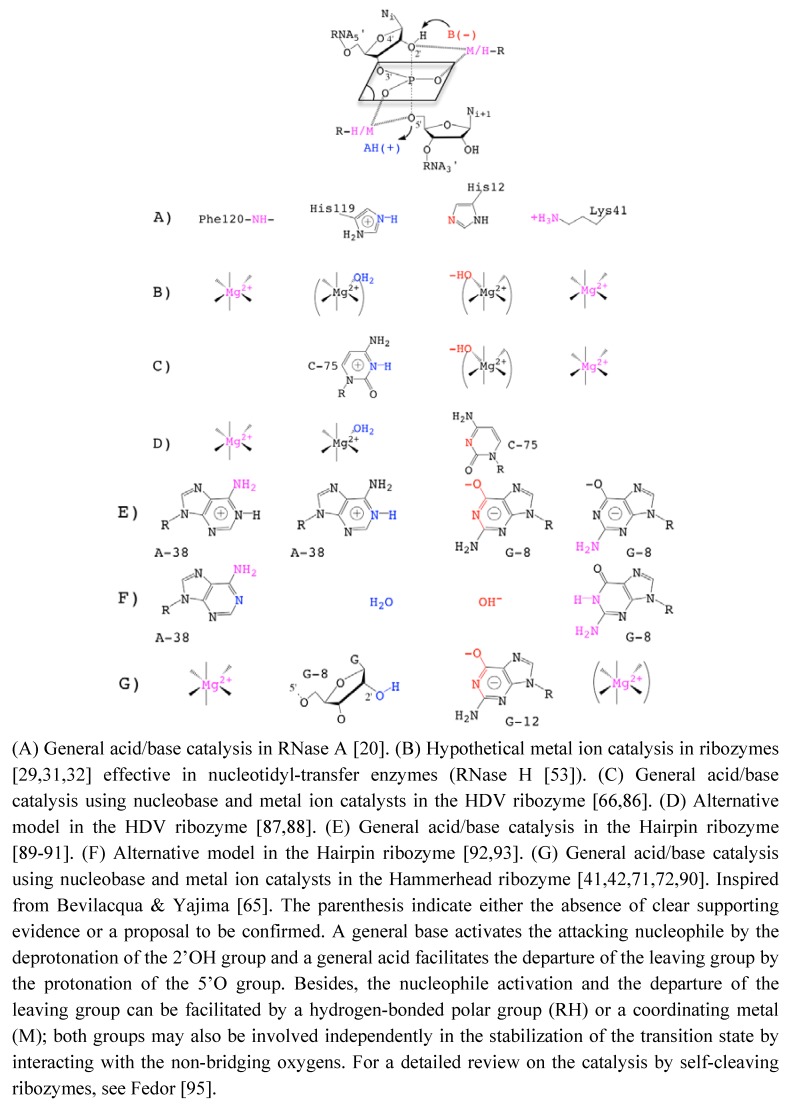
Catalytic Strategies in the Transphosphoryl Reaction of RNase A and Self-Cleaving Ribozymes.

The role of divalent cations in the HHRz catalysis was first questioned when monovalent ions were shown to preserve some catalytic activity [24]. The fact that divalent cations such as Mg^2+^ could be replaced by Na^+^, Li^+^ or NH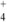 [24,38,72] without abolishing the catalytic activity was taken as an evidence of the minor or strictly structural role of divalent cations. Those metals would just play a nonspecific role in charge screening (zero-metal mechanism) [73]. In addition, the remaining catalytic activity in the absence of divalent metal ions in various other self-cleaving ribozymes [24,74] was suggesting alternative catalytic strategies that would rely on nucleobases as general acid/base catalysts [65,75] (Figure 3).

In protein enzymes, a major contribution to the catalytic power comes from the electrostatic preorganization of the catalytic site [76] which contributes significantly to lower the free energy barrier of activation [77,78]. If this also stands for RNA enzymes, we can assume that the poor catalytic activity in presence of monovalent ions actually reflects a major role for divalent cations in the optimization of the catalytic power of HHRz. Then, it is still unclear whether the contribution of divalent cations just comes from the charge density and the ion mobility or whether it also results from other physico-chemical properties related to specific interactions with RNA. Also, the coordination repertoire of Mg^2+^ might be more extended than the usual hexacoordinated form as suggested by various theoretical studies where anionic ligands trigger a coordination change [32,79,80]. Thus, the divalent metal ions might also function in ribozymes as reservoirs of "activated" water molecules acting as proton donor or acceptor [81]. They could also induce conformational changes in the folded RNA of self-cleaving ribozymes [59,82,83] and among them in the HHRz [58].

In the HHRz, recent data largely support the implication of one or several nucleobases as general acid-base catalysts [39,84]. Using nonstandard nucleotides carrying or not an imino proton at the N1 position, the cleavage rate profiles were shown to be differentially pH-sensitive [39]. The cleavage rate was more affected in the HHRz variants at G8 and G12 positions in which the imino proton was removed. Accounting for the p*K_a_* of the different nonstandard nucleotides, G8 and G12 were considered as potential general acid/base catalysts [39]. However, assigning a specific role to nucleobases is also challenging because of their multiplicity of roles (Figure 3 G). For example, the pH-sensitive cleavage rate of G8 and G12 variants may correlate with a direct implication in the chemical reaction. Alternatively, it might relate to a change in the RNA structure due to weakening some base-pairings (G12:A9, G8:A13 or other transient base-pairings involving G12 or G8). Without consistent structural information, it was difficult to speculate on a "specific" or "nonspecific" role for both G8 and G12 even though the profile for the variant carrying an imino proton (G to I) was different. Indeed, the pH profile was more affected in G12 variants, suggesting a distinct behavior at this position; folding interferences at G8 but not at G12 were also consistent with a more "specific" role of G12 [39]. The recent structure determination of an extended HHRz [42] reconciles structural and old biochemical data [85]. It also provides a detailed picture about the positions and possible roles of G8 and G12 in the catalytic pocket consistent with a general acid/base catalysis. In the proposed reaction mechanism, the N1 of G12 and the 2’OH of G8 are responsible for a general acid/base catalysis by deprotonating the 2’OH nucleophile and protonating the 5’-O leaving group, respectively [42]. The role of G12 as a general base is further supported by experimental data that reveal alkylation of G12-N1 by a bromoacetamide probe as result of a N-nucleophilic attack on a substrate analog [40] (Figure 3G).

### 2.3. Metals & Nucleobase in the HHRz Catalysis

The recent experimental evidences supporting a general acid/base model of catalysis partially eclipsed the persistent roles of divalent cations in both the minimal and extended HHRz. A higher concentration of metal ions is required to properly fold the minimal HHRz and induce a conformational change towards the transition state [58]. The lower concentration required to fold the extended HHRz may suggest that most of the metal binding sites are optional in the folded form of the extended HHRz. However, at least two putative metal binding sites were then shown to be conserved in both the minimal and extended HHRz [36]. Both HHRz also happen to share a common dynamic reaction mechanism [96]. Later, a comparative study on the influence of different metals suggested that a metal ion takes a an active part in the catalysis although it was excluded from acting as a general base in the reaction [37,97]. Instead, a metal ion was proposed to make a direct coordination with a nucleobase that would act as the general acid suggesting that the reaction mechanism may proceed via a general acid/base catalysis assisted by a metal cofactor [97]. A similar study carried out on metal ion specificities for folding and cleavage activity also suggested the presence of a critical bound metal acting as a structural metal. It could (re)shape the catalytic site, induce a shift in the equilibrium between inactive and active conformations, or stabilize the negative charge in the transition state [64]. More recently, a functional study of the general acid catalysis mechanism came also to the conclusion that a metal ion is involved [41] in making the 2’OH of G8 more acid to be transferred to the 5’O leaving group and stabilizing the transition state. Furthermore, a metal ion was proposed very recently to interact both with the 2’-OH nucleophile and the pro-*R* oxygen of the scissile phosphate group suggesting the presence of a ‘specific’ catalytic ion during the nucleophile activation [94] (Figure 3G). 

Taken together, these recent data indicate that the structural and catalytic roles of metal ions and nucleobases are both fuzzy and intricate. So, it is challenging to determine what metal ions and nucleobases do exactly in the catalysis (in the activation of the 2’OH nucleophile, in the transition state stabilization and in the protonation of the 5’O leaving group). Indeed, both nucleobases and metal ions likely have multiple roles as electrostatic catalyst and/or as general base on the one hand and as electrostatic catalyst in the nucleophile activation and/or as general base on the other hand (Figure 3C-G). In the case of the hairpin ribozyme, it is not clear yet whether the nucleobases contribute more significantly to the catalysis as general acid/base or as electrostatic catalysts [89,90,91,92,93] (Figure 3E-F). As pointed out in a review on HHRz structures, “When to believe what you see" [98], the X-ray structures available for various HHRz are "only snapshots of dynamic processes ...”. Thus, what we can see may not be fully relevant from the functional point of view while what we do not see, like metal ions, may be relevant. 

**Figure 4:**
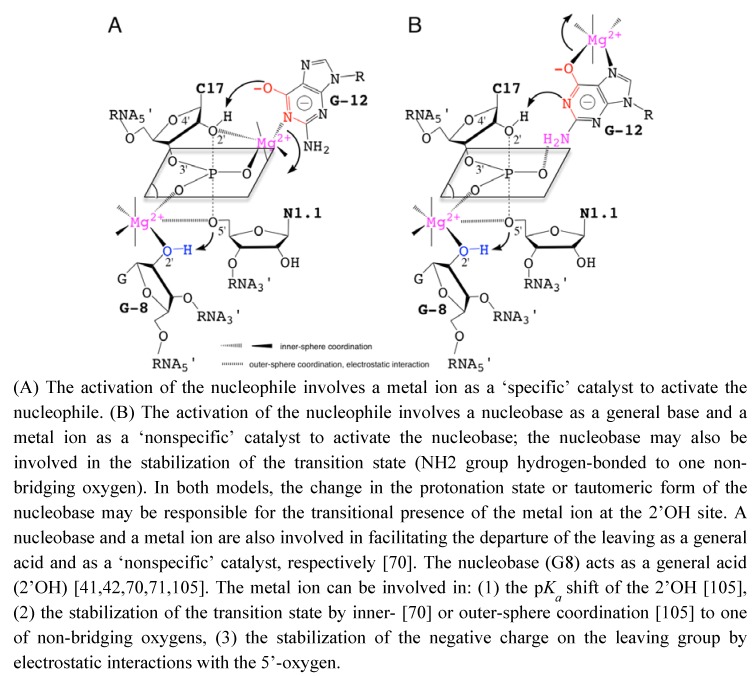
Hypothetical Models for the Nucleobase-Metal Catalysis in the Hammerhead Ribozyme.

To reconcile all the recent data described previously, hypothetical models of catalysis are proposed based on the participation of metal ions and nucleobases as ‘specific’ or ‘nonspecific’ catalysts. The roles of metal ions in the catalytic site are also extrapolated from data on nucleobase-p*K_a_* shifts due to metal binding [61,99] (Figure 4). These models are more specifically inspired from the recent experimental work by Osborne *et al.* [94] on the presence of a metal ion close to the 2’OH nucleophile and from the theoretical studies by Lee *et al.* [100,101] on the role of a metal ion close to the leaving group. G12 was proposed to act as a specific catalyst in the nucleophilic activation [40]. However, the role of G12 is mostly supported by the N1-alkylation of the nucleobase.

On a substrate analog chemically modified where the 2’-OH group is replaced by a bulky 2’-bromo-acetamide. Thus, the reaction mechanism on the substrate analog may not reflect exactly how the natural substrate is processed and may exclude the possible implication of a metal ion close to the 2’-OH of C17. A metal ion could act with the nucleobase in a cooperative way to facilitate the activation of the 2’-OH nucleophile either: (1) as a specific catalyst by coordinating the 2’-oxygen (Figure 4A), or (2) as a nonspecific catalyst by stabilizing an activated form of G12 (Figure 4B).

Additionally, the nucleobase could be involved in the stabilization of the pentacoordinated phosphate group. The lower catalytic activity in variants carrying a 7-deaza modified guanine at G12 may result from the presence of a metal ion as nonspecific catalyst [41]. Actually, the results from molecular simulation suggested that the metal ion initially bound to A9/G10.1 (C-site, Figure 2A) would migrate to G8 (bridging-site, Figure 2A). The migration would occur in an concerted way with the deprotonation of the 2’-OH through a large-scale conformational rearrengement [100]. This may indicate that the first metal ion bound to G12 during the nucleophile activation is released before the binding of the second metal to G8. The results obtained by Thomas and Perrin [41] and Lee and York [101] are both consistent with the 2’-OH group of G8 acting as general acid catalyst in a cooperative way with a metal ion. Its proximity with the 5’-O leaving group presupposes the metal also contributes electrostatically to facilitating the departure of the leaving group. 

Up to now, no model of nucleobase-metal cooperativity has been proposed in ribozymes but it was recently suggested by Schnabl and Sigel [61] from the data published on the modulation of nucleobase p by the ionic environment [102]. Those two models (Figure 4) may be supported to a different extent by functional group modifications at G8 and G12 positions [41,86]. The substitutions of G12 by modified 7-deaza/7-methyl and/or 6-thio analogs and their impact on the catalytic rate may give some hints on metal coordinations (Figure 4B). Both models may not totally exclude each other but exist at different time frames, the two static chemical representations are given for convenience as the final state before cleavage. The metal acts by positioning G12 within the catalytic site (Figure 4A) or by activating the nucleobase as catalyst (Figure 4B). In the second model, the metal may also be carried by G12 on its hoogsteen face during the conformational change, in a similar way to the suggested migration from the C-site to the bridging-site (Figure 2A) [68]. So, both representations might be partially true considering the dynamics within the catalytic pocket. Experimental methods designed to study the RNA dynamics and fast events should be helpful to characterize transient intermediates. NMR spectroscopy and X-ray crystallography methods have been successful to identify tight or loose metal binding sites [23,55,56], they may be used to revisit the metal binding sites in the extended HHRz. Metals coordinated to the bridging oxygens of phosphate groups can be detected by the measurement of isotope effects [19] using for example: a labeled 2’O-cytidine at C17 and a 5’O-guanidine at G8. Similarly, other techniques can provide information on metals bound to nucleobases [103] by measuring for example at G12: J-couplings using a N7-guanidine or chemical shifts perturbations using a C8-guanidine. Experimental data obtained using these techniques may reveal new metal binding sites as proposed here (Figure 4) or/and confirm the metal coordinations proposed previously [41,67,68,95]. Recently, a combination of NMR and XAS disclosed a new metal binding site within the ribonuclease P ribozyme [104], it is thus a promising approach to discover extra metal binding sites in the HHRz. Quantum mechanical/molecular mechanical (QM/MM) simulations should also help to identify the more relevant reaction mechanisms and reaction paths as well as the major contributions to the catalytic rate as suggested by recent studies [67,68,101,105]. Extended studies to new models and HHRz variants may have a predictive value.

### 2.4. Possible Evolution and Origin of the Catalytic Power in the HHRz

Beyond the reaction mechanism, what we can see close to the cleavage site may not be what most contribute to the catalytic power of the ribozyme. Recent QM/MM studies performed on the hairpin ribozyme suggest that the two nucleobase catalysts (Figure 2E-F) [89,90,91] may contribute to lowering the activation barrier mostly by creating a favorable electrostatic environment rather than acting as proton donor and acceptor [93]. So, we can assume that the electrostatic preorganization of the catalytic site is a major contribution to the catalytic power of ribozymes. Let us examine separately the chemical processes involved in the catalysis: the activation of the 2’OH nucleophile, the stabilization of the transition state and the departure of the leaving group. Recent experimental data suggest the presence of a metal ion coordinating the pro-R oxygen of the scissile phosphate group that may facilitate the nucleophile activation [94]. Furthermore, QM and DFT calculations performed on a minimal active site model [32] suggest that a nucleobase is not a better general base than a metal hydroxide to activate the 2’OH nucleophile (Chval & Leclerc, to be published). On the other hand, a metal-coordinated nucleobase could be a more efficient catalyst via ligand-p*K_a_* shift [99]. The presence of a metal coordinated to one of the non-bridging oxygens though not coordinated to the 5’O leaving group [41,94] would contribute to the stabilization of the transition state via charge-charge interaction. This same metal ion, also coordinating the 2’OH of G8, would shift the p*K_a_* of the 2’OH and thus facilitate the protonation of the 5’O leaving group [41]. Whether one or two metal ions are present at the same time in the catalytic site is unclear but cooperative effects might be involved in the latter case [32] (Figure 4). All together, the data suggest the metal ions are ‘specific’ or ‘nonspecific’ catalytic ions; pushing more: one may consider those metal ions as obligate cofactors bound to nucleobases. The nucleobases would contribute energetically to the catalytic rate more by the reorganization of the catalytic site than by their direct participation in the chemical processes.

**Figure 5:**
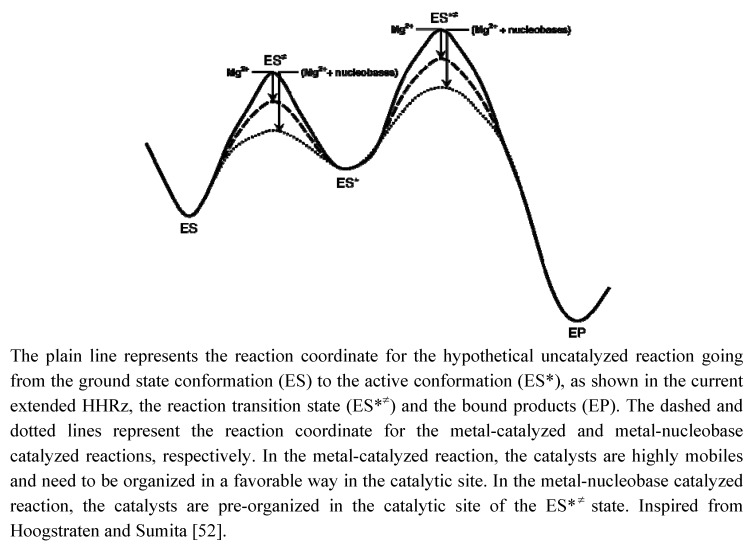
Schematic reaction coordinate indicating the possible roles of Mg^2+^ and nucleobases as catalysts in ‘minimal’ and ‘extended’ ancestor variants of the HHRz.

The active conformation of the HHRz requires to reshape completely the catalytic pocket via a major conformational change. Nucleobases are the main chemical moieties of nucleotides responsible for the base-pairings that may stabilize different transient conformational intermediates. Because of their affinity for metals, the nucleobases could also reorganize the catalytic site by trapping metal ions at specific sub-sites and create an electrostatic preorganization that would lead to lowering the free energy of activation (Figure 5). Thus, we can imagine that the minimal HHRz is reminiscent to some ancestral variant behaving as a true metalloenzyme where metal ions used to play both structural and catalytic roles. The small size of the ribozyme would not allow the RNA to fold directly into the active conformation once the enzyme and substrate components are paired. The need for a major conformational change would then preclude the selection for tight metal binding sites but instead maintain loose binding sites and highly mobile metal ions. The migration of metal ions could then induce conformational changes directly or indirectly by modulating the p*K_a_* of nucleobases. During the evolution from a ‘minimal’ to an ‘extended’ ancestor, the RNA extensions in H1 would have allowed a preorganization of the HHRz more favorable to the transition towards the active conformation. The phylogenetic conservation of G8 and G12 would thus reflect the selection of nucleobases at two positions essential for the electrostatic preorganization of the catalytic site by base-pairing or by trapping metal ions.

## 3. Conclusions

Since its discovery several decades ago, the HHRz gradually exposed some of the major forces operating in RNA catalysis for one the most studied ribozymes, but it has not disclosed yet all of its secrets. A new way of looking at this ribozyme based on evolutionary and dynamic considerations may help to go further in the validation of more completed models of catalysis. Although RNAs have a more restricted residue library than proteins for catalysis, their chemical repertoire appears to be more extended than previously thought. In fact, the RNA catalysis is based on a more context-dependent chemical repertoire and thus more difficult to decipher but also more challenging to understand.
